# Acute Kidney Failure: Current Challenges and New Perspectives

**DOI:** 10.3390/jcm12103363

**Published:** 2023-05-09

**Authors:** Shanshan Chen, Yupei Li, Baihai Su

**Affiliations:** Department of Nephrology, West China Hospital of Sichuan University, Chengdu 610041, China; 2016181622062@stu.scu.edu.cn

Acute kidney failure, also called acute kidney injury (AKI), is defined by a sudden loss of kidney function that is conventionally determined on the basis of increased serum creatinine levels and reduced urinary output. AKI is a complex syndrome with high morbidity and mortality rates. AKI can not only lead to short-term adverse outcomes, but also leave survivors at risk for chronic kidney disease (CKD), cardiovascular events, and kidney cancer. There is also a high incidence of COVID-19-related AKI, synonymous with its global outbreak. Therefore, AKI affects the quality of life of survivors and results in heavy healthcare burdens.

At present, it is still difficult to diagnose AKI. First, AKI may be asymptomatic in its early stages because clinical signs depend on the degree of renal impairment. Second, the glomerular filtration rate (GFR) is a key marker that is used to evaluate renal function, but there are currently no available tools to monitor real-time GFR. Serum creatinine and urine volume are used to assess the changes in GFR in current clinical practice, but they are neither sensitive nor specific. An increase in serum creatinine levels can only be detected when there is at least a 50% loss of kidney function in previously healthy patients [1]. In addition, several factors can affect serum creatinine levels. For example, patients with sepsis may experience a decrease in creatinine production, and then a drop in creatinine may occur, while the high baseline serum creatinine levels of patients with pre-existing CKD may lead to misclassification errors. The volume expansion and loss of muscle mass in critically ill patients may also lower the concentration of serum creatinine. In comparison to serum creatinine, urine output is extremely sensitive to overall volume status. Therefore, neither serum creatinine nor urine output can reflect the timely development of kidney injury. As stated in the Kidney Disease Improving Global Outcomes (KDIGO) guideline [2], AKI is a clinical diagnosis that requires further clinical evidence. Patient history, blood tests, urine microscopy, renal imaging, and renal biopsy are useful in providing information on the causes of AKI, but all of these methods are controversial and ineffective in diagnosing early AKI. It is generally accepted that the stage and duration of an episode of AKI are strongly associated with long-term outcomes, including survival and kidney recovery. Specifically, AKI patients with a transient reduction in excretory function may fully recover without causing irreversible nephron loss, whereas AKI with extensive nephron damage may progress to post-AKI CKD and even end-stage kidney disease. Hence, early detection and diagnosis are extremely significant for a prognosis of AKI.

The clinical management of AKI is also challenging due to its complex pathophysiological mechanisms caused by multifactorial etiology and comorbidities. The key principle of AKI management is to treat the underlying causes of AKI and mitigate further kidney damage. However, as mentioned earlier, the causes of AKI are complicated and are often identified too late by clinicians. By then, kidney damage may have already occurred, and there is unfortunately no targeted pharmacological approach. Furthermore, patients with AKI may develop many complications, such as fluid, electrolyte, and acid–base imbalances. Therefore, symptomatic and supportive therapies are the primary clinical strategies. Fluid management is a significant component in the prevention and treatment of AKI. Isotonic crystalloids play a role in the initial management for the correction of intravascular hypovolemia, but more high-quality data and clinical trials are required to verify this hypothesis. Moreover, it is still controversial or evidence-limited in other management strategies, such as the most effective vasopressor agent, the use of diuretics, the control range of blood glucose, and the optimal amount of protein supplementation [2]. Renal replacement therapy (RRT) is inevitable and life-saving in severe AKI. Refractory hyperkalemia, metabolic acidosis, volume overload, or signs of uremia in AKI patients are the most accurate indications of RRT initiation, but there is substantial controversy surrounding the optimal timings to start RRT in patients without severe complications [3]. There are still ongoing debates surrounding whether intermittent or continuous RRT should be used and when RRT should be stopped [4,5].

Despite these current challenges, there have been many advances in the diagnosis and management of AKI. A body of evidence from preclinical and clinical studies shows that the use of novel biomarkers has the potential to significantly improve AKI diagnosis and management. Novel biomarkers have been shown to change earlier than serum creatinine concentrations and can be broadly categorized as stress markers, damage markers, and functional markers. Accordingly, a consensus statement proposed that damage markers should be combined with functional biomarkers and clinical information to expand and refine the KDIGO definition of AKI [6]. The early detection of AKI using biomarkers may help clinicians take timely interventions before irreversible AKI injury occurs, consequently improving the outcomes. Additionally, new biomarkers may be helpful when considering the withdrawal of RRT based on conventional biomarkers [7]. However, most of these kidney biomarkers are still in the clinical trial stage, and their adoption in clinical practice is slow. In addition to biomarkers, attempts are being made to diagnose AKI through the use of electronic alerts and machine learning [8]. The electronic AKI alerts collect data from electronic health record systems and then automatically decide whether to trigger electronic alerts based on changes in the key variables. Machine learning is based on large datasets as well, but it also focuses on building predictive models that can improve the accuracy of an AKI diagnosis. Clearly, data collection is a barrier to the application of electronic alerts and machine learning. With the development of technology, new detection methods, such as renal contrast-enhanced ultrasonography, multiparametric MRI, optical probes, and electrochemical immunosensors, also seem to provide information about renal microcirculation and improve the accurate and timely detection of biomarkers [9].

From a treatment perspective, in addition to the traditional role of RRT, such as maintaining a fluid and electrolyte balance, RRT with extracorporeal hemoadsorption can be applied to remove inflammatory mediators. Although the pathogenesis of AKI is incompletely understood, the dysregulation of an immune response is considered to be one of the pathological mechanisms of sepsis and COVID-19-associated AKI. Theoretically, the removal of inflammatory mediators in the early phase to achieve immune homeostasis can relieve the dramatic systemic effects of severe infection [10]. Recently, several hemoadsorption technologies, such as high-cut-off membranes, the Ultraflux EMIC2 filter, the oXiris hemofilter, polymyxin B hemoadsorption, CytoSorb, and HA380, have been studied in clinical practice [11]. Multiple clinical trials have reported encouraging results, but there is insufficient amount of evidence to demonstrate any survival benefits [12]. In addition, some key issues, including the selection of optimal patients and filters, the correct initiation time and duration, and the definition to evaluate efficacy, all remain elusive [12,13]. Beyond inflammation, it is generally accepted that a decrease in renal perfusion is the primary mechanism for most AKI cases. The resulting renal hypoxia and ischemia promote an increase in reactive oxygen species (ROS). There is a considerable amount of evidence which demonstrates that increased ROS and decreased antioxidants can be detected in AKI [14]. Thus, scavenging excessive ROS may be a novel and specific treatment for AKI. Numerous antioxidants, such as vitamin C, antioxidant enzyme mimetics, heme oxygenase-1, and N-acetylcysteine, have been tested [15]. With the rapid development of nanotechnology, various nanomedicines with ROS scavenging actions have also been synthesized to ameliorate AKI [14]. The efficacy of antioxidants and nanomedicines has been proven in vitro and in animal models. However, it is worth noting that the translation of antioxidants to human AKI has shown little benefits, and research on antioxidant nanomedicines in AKI is still in its infancy [14,15]. Finally, stem-cell-based therapy, gene therapy, and artificial kidneys may also be effective for AKI [16,17,18].

In conclusion, although there are some challenges in the current diagnosis and management of AKI, the development of methods and technologies is helping to create opportunities for the early detection and treatment of AKI. With an improved understanding of the pathophysiology, targeted therapies for AKI may also emerge and be applied in clinics. With this Special Issue, we hope to provide an overview of new perspectives for AKI diagnosis and management.
